# Association between neuroticism and cosmetic mammoplasty: Results from a bidirectional Mendelian randomization study

**DOI:** 10.1097/MD.0000000000047580

**Published:** 2026-02-20

**Authors:** Qiyu Liu, Li Yu, Xueyao Cai, Yuchen Cai, Wuliang Diao

**Affiliations:** a Department of Plastic Surgery, Shenzhen People’s Hospital (The Second Clinical Medical College, Jinan University; The First Affiliated Hospital, Southern University of Science and Technology), Shenzhen, Guangdong, China; b Department of Plastic and Reconstructive Surgery, Shanghai Ninth People’s Hospital, Shanghai Jiao Tong University School of Medicine, Shanghai, China; c Department of Plastic Surgery, Affiliated Hospital of Guizhou Medical University, Guiyang, Guizhou, China.

**Keywords:** cosmetic mammoplasty, Mendelian randomization, neuroticism

## Abstract

Observational studies have suggested associations between neuroticism and the tendency to undergo cosmetic mammoplasty, but their causal relationships remain unclear. Our study aimed to assess the causal associations between neuroticism and cosmetic mammoplasty using a bidirectional 2-sample Mendelian randomization method. Genetic associations with neuroticism (n = 329,821) and cosmetic mammoplasty (n = 462,933) were identified in genome-wide association studies of European ancestry. The inverse-variance weighted (IVW) was designated as the primary model for data analysis, supplemented with weighted median, Mendelian randomization-Egger, weighted mode, and simple mode method. Genetically determined neuroticism was positively associated with the incidence of cosmetic mammoplasty (IVW odds ratio = 1.002, *P* = .019). Reversely, predisposition to cosmetic mammoplasty genetically increased the risk of neuroticism (IVW odds ratio = 21.041, *P* = 2.05 × 10^–3^). Our results suggested a bidirectional causal relationship between neuroticism and cosmetic mammoplasty. These findings may highlight the potential influence of psychological factors on patients undergoing cosmetic surgery.

## 1. Introduction

Neuroticism refers to a stable personality trait that assesses individual differences to experience negative emotions and emotional instability. As one of the most widely studied psychological dispositions, it has far-reaching implications for public health.^[1]^ Highly neurotic people are more susceptible to psychiatric illness and tend to have psychological stress. Previous studies have indicated that neuroticism has a strong connection with a large spectrum of mental disorders.^[2,3]^ Moreover, accumulating evidence has suggested the link between neuroticism and the occurrence of various adverse health outcomes, including obesity, cardiovascular disease (CVD), and surgical complications.^[4–6]^

Cosmetic breast surgery plays a critical role in the psychological and physical well-being of patients.^[7]^ Although extensive research has been conducted to explore the role of personality traits in cosmetic surgery, evidence linking neuroticism to cosmetic mammoplasty remains scarce and inconsistent to date. In previous studies, no preoperative neurotic symptom was found in patients preparing for cosmetic mammoplasty.^[8]^ In contrast, some authors suggested a low degree of association between neuroticism and the tendency to undergo breast augmentation surgery.^[9,10]^ Interestingly, a relatively high level of neuroticism was also found in postoperative women with breast implants.^[11]^ Since observational studies are prone to confounding bias or reverse causation, the actual causal relationship between neuroticism and cosmetic mammoplasty remains to be determined.

As an alternative method, Mendelian randomization (MR) uses single nucleotide polymorphisms (SNPs) as genetic variants to examine the causal association of exposures with outcomes.^[12]^ Because SNPs are randomly allocated at meiosis and cannot be influenced by sociodemographic or behavioral factors, their use as instrumental variables (IVs) can produce results that are independent of confounders or reverse causality.^[13]^ Herein, we used the MR approach to investigate the causal relationship between neuroticism and cosmetic mammoplasty based on publicly available genome-wide association studies (GWAS).

## 2. Methods

### 2.1. Study design

The 2-sample MR approach was adopted using the publicly available GWAS summary data with ethical approval obtained in the original studies. SNPs with conflicting alleles between the neuroticism and cosmetic mammoplasty cohorts were excluded. A forward MR model was established to assess the causal associations of neuroticism with cosmetic mammoplasty. Subsequently, a reverse MR analysis was conducted to examine the causal effect of cosmetic mammoplasty on neuroticism.

### 2.2. GWAS data source and IVs selection

All patient data were collected from the online IEU Open GWAS database (https://gwas.mrcieu.ac.uk/datasets/) of European ancestry. Sample sizes were 329,821 and 462,933 for neuroticism (dataset: ebi-a-GCST005232) and cosmetic mammoplasty (dataset: ukb-b-10617), respectively. The neuroticism phenotype in the source GWAS was derived from scores on the 12-item Eysenck Personality Questionnaire—Revised Short Form (EPQ-R-S), a validated measure of emotional instability administered within the UK Biobank cohort.^[14]^ Although this scale originates from Eysenck 3-factor (PEN) model of personality, extensive genetic research has demonstrated that the neuroticism construct it captures is genetically virtually identical to the neuroticism dimension within the Big 5 (five-factor) model, with a genetic correlation approaching 1.0.^[14]^ SNPs reaching the genome‐wide significance (*P* <1 × 10^−5^) were designated as IVs for further analysis. Linkage disequilibrium was applied to test genetic linkage among selected SNPs on criteria of *r*^2^ <0.001 with a kb = 10,000 window size.

### 2.3. Bidirectional MR analysis

The inverse-variance weighted (IVW) method was designated as the primary analysis algorithm to infer causality, followed by sensitivity analyses including weighted median, MR-Egger, weighted mode, and simple mode.^[15]^ IVW is the most common approach in 2-sample MR, which provides the best unbiased estimate assuming IVs are valid and without pleiotropy.^[12]^ The odds ratio (OR) of IVW was defined as the primary outcome, indicating the odds of an outcome influenced by exposure. The MR-Egger intercepts are used to evaluate possible directional pleiotropy effect across genetic variants.^[16]^ All MR analyses were performed using the R software (version 4.1.2) with the “TwoSampleMR” package (version 4.1.1). A *P*-value <.05 was deemed statistically significant in the bidirectional MR analyses.

## 3. Results

### 3.1. Causal effects of neuroticism on cosmetic mammoplasty

**Figure 1.**
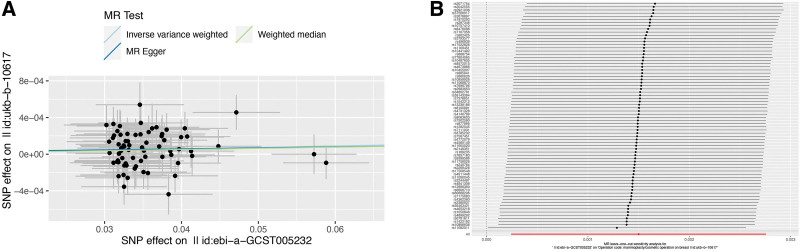
Mendelian randomization of neuroticism on cosmetic mammoplasty. (A) Scatter plot, and (B) leave-one-out sensitivity analysis of the genetic associations between neuroticism (exposure) and cosmetic mammoplasty (outcome).

In total, 72 independent SNPs were selected as IVs to analyze the causal effect of neuroticism on cosmetic mammoplasty. The consistent direction of the scatter plot suggested that genetically determined neuroticism had a strong and positive association with the incidence of cosmetic mammoplasty (IVW OR = 1.002, *P* = .019) (Fig. 1A and Table 1). In MR-Egger regression, the intercept indicated no horizontal pleiotropy in the MR model (Fig. 1A). In addition, the leave-one-out analyses were employed, confirming that no individual SNP drove these results (Fig. 1B).

**Table 1:** Causal associations of the neuroticism IVs with cosmetic mammoplasty.

MR method	SNP (n)	Beta	OR	95% CI	*P*-value
IVW	72	0.0015	1.002	1.000, 1.003	**.019**
WM		0.0013	1.001	0.999, 1.003	.180
MR-Egger		0.0011	1.001	0.992, 1.010	.812
Weighted mode		0.0005	1.001	0.996, 1.005	.802
Simple mode		0.0015	1.002	0.997, 1.006	.481

CI = confidence interval, IVs = instrumental variables, IVW = inverse-variance weighted, MR = Mendelian randomization, OR = odds ratio, SNP = single nucleotide polymorphism, WM = weighted median method.

### 3.2. Causal effects of cosmetic mammoplasty on neuroticism

**Figure 2.**
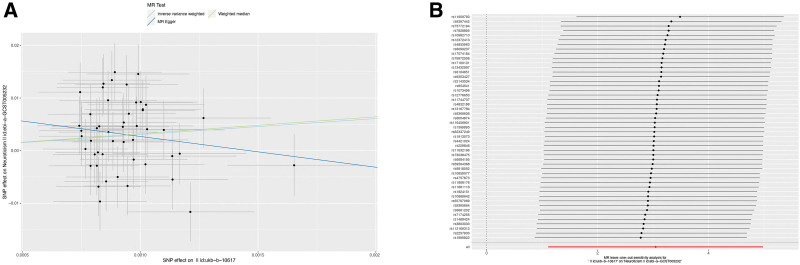
Mendelian randomization of cosmetic mammoplasty on neuroticism. (A) Scatter plot, and (B) leave-one-out sensitivity analysis of the genetic associations between cosmetic mammoplasty (exposure) and neuroticism (outcome).

For reverse MR, a total of 49 independent SNPs were used as IVs to explore the causal association of cosmetic mammoplasty with neuroticism. It was found that genetical predisposition to cosmetic mammoplasty increased the risk of neuroticism (IVW OR = 21.041, *P* = 2.05 × 10^–3^) (Fig. 2A, Table 2 and Table S1–2, Supplemental Digital Content, https://links.lww.com/MD/R371). The MR-Egger intercepts showed no directional horizontal pleiotropy, while the leave-one-out analysis supported that the overall MR estimates were not influenced by a single SNP (Fig. 2A and B and Fig. S1, Supplemental Digital Content, https://links.lww.com/MD/R370).

**Table 2:** Causal associations of the cosmetic mammoplasty IVs with neuroticism.

MR method	SNP (n)	Beta	OR	95% CI	*P*-value
IVW	49	3.0465	21.041	3.034, 145.906	**2.05 × 10^–3^**
WM	–	3.2139	24.876	1.904, 324.957	**.014**
MR-Egger	–	−5.8484	2.88 × 10^–3^	3.50 × 10^–8^, 237.731	.316
Weighted mode	–	3.6520	38.552	0.124, 11989.923	.218
Simple mode	–	4.3701	79.050	0.238, 26216.967	.147

CI = confidence interval, IVs = instrumental variables, IVW = inverse-variance weighted, MR = Mendelian randomization, OR = odds ratio, SNP = single nucleotide polymorphism, WM = weighted median method.

## 4. Discussions

In this large-scale MR analysis, we examined the bidirectional causal association between neuroticism and cosmetic mammoplasty in large-population longitudinal GWAS data. We observed that genetically determined neuroticism is positively associated with the incidence of cosmetic mammoplasty. Reversely, predisposition to cosmetic mammoplasty genetically increased the risk of neuroticism.

Cosmetic surgery plays a major role in psychological well-being of patients, corresponding with a trend in modern society to evaluate a person based on one’s physical appearance.^[17]^ Despite a high prevalence of cosmetic mammoplasty around the globe, studies have been missed on evaluating the psychological profiling of these individuals. Previous studies have been scarce and inconsistent on the relationship between neuroticism and cosmetic mammoplasty. The majority of these studies indicated null or weak positive relationships, but remained unclear about their bidirectional causal associations.^[18]^ To the best of our knowledge, this is by far the largest study investigating the causal relationship between neuroticism and cosmetic mammoplasty, providing a greater statistical power to uncover causal associations. Our MR study design exclude the interference of confounding bias and was robust to multiple sensitivity analyses for outliers, reverse causality, and horizontal pleiotropy. Taken together, these findings revealed a significantly positive bidirectional causal association between neuroticism and cosmetic mammoplasty.

Several explanations may justify the underlying bidirectional links between neuroticism and cosmetic mammoplasty. Neurotic individuals, who are often accompanied by strong negative emotions, have an increased chance of body dissatisfaction.^[19]^ Previous studies have shown a positive effect of cosmetic mammoplasty on patients’ self-reported quality of life.^[20]^ Therefore, it is possible that individuals with a higher neuroticism level are more likely to perform cosmetic surgery to compensate for their lower life satisfaction compared to their healthy counterparts. Meanwhile, our reverse MR showed a positive effect of cosmetic mammoplasty on neuroticism levels. According to various published works, a number of postoperative cosmetic surgery patients experience little or no extent of increase in body image and self-esteem evaluation.^[21,22]^ It is therefore expected that cosmetic surgery may produce negative psychological effects in certain patients due to a lesser improvement in body image, with a long-term adverse effect that could lead to neurotic symptoms. While our study demonstrated the bidirectional relationship between neuroticism and cosmetic mammoplasty, the exact mechanism for their association needs to be elucidated by future research.

Our study has several notable strengths. First, this is the initial study to investigate the bidirectional causal relationship between neuroticism and cosmetic mammoplasty through a 2-sample MR analysis. The large sample size provides a higher level of evidence than previous observational studies. Second, the present results were confirmed in sensitivity analyses, which minimize confounding bias and provide a strong causal association. However, our MR study also has some unavoidable limitations. First, the results may not be generalized to other populations because our study focused on individuals of European ancestry. Future studies are warranted to validate our findings in different regions. Second, because the neuroticism data in GWAS was self-reported at baseline, the misclassification of certain patients may affect the magnitude of associations. Third, we were unable to assess the degree or severity of neuroticism because no stratification data were included in GWAS. Further analyses should be carried out when more detailed datasets become available. Fourth, the GWAS data for cosmetic mammoplasty did not distinguish between different motivations for surgery, such as purely aesthetic enhancement versus postmastectomy reconstruction. The estimated causal effects represent an average across these potentially heterogeneous groups, and future studies with more detailed phenotypic data are needed. Fifth, in the reverse MR analysis, the IVs index genetic liability to cosmetic surgery, not the direct physiological or psychological impact of the surgical procedure itself. This means our estimates reflect the effect of a lifelong genetic predisposition, which may differ in magnitude from the effect of undergoing surgery. Finally, while we focused on neuroticism due to its prominent theoretical and empirical links to body image and health behaviors, other personality traits (e.g., extraversion, conscientiousness) may also influence the propensity for cosmetic surgery. Future multivariate genetic studies incorporating a broader personality spectrum are warranted.

## 5. Conclusion

In summary, our study suggested a bidirectional causal relationship between neuroticism and cosmetic mammoplasty. These findings may highlight the potential influence of psychological factors in patients undergone cosmetic surgery. Further large-scale and longitudinal studies are warranted to determine the specific pathogenic mechanisms.

## Acknowledgments

We gratefully acknowledge the IEU Open GWAS project for providing GWAS data resources used in this study.

## Author contributions

**Conceptualization:** Xueyao Cai, Wuliang Diao.

**Formal analysis:** Wuliang Diao.

**Investigation:** Xueyao Cai.

**Methodology:** Li Yu, Wuliang Diao.

**Resources:** Xueyao Cai.

**Supervision:** Li Yu.

**Writing – original draft:** Wuliang Diao.

**Writing – review & editing:** Qiyu Liu, Li Yu, Yuchen Cai.

## Supplementary Material
