# Supplementary material for: Association between neuroticism and cosmetic mammoplasty: Results from a bidirectional Mendelian randomization study
Source: Medicine (Baltimore). 2026 Feb 20;105(8):e47580. doi: 10.1097/MD.0000000000047580 (PMC12928946; doi:10.1097/MD.0000000000047580)

**Supplementary Figure 1.** Mendelian randomization (MR) for the causal effect of cosmetic mammoplasty on neuroticism. (A) Scatter plot for associations between genetic variants associated with cosmetic mammoplasty and their effects on neuroticism. The dots represent the effect size of each SNP on cosmetic mammoplasty (x-axis) and neuroticism (y-axis). (B) Funnel plot of the genetic risk of cosmetic mammoplasty on neuroticism. (C) Forest plot for genetic risk of cosmetic mammoplasty on neuroticism. The red dots and lines indicate aggregate MR effect estimates and confidence intervals (CI) as indicated by the MR-Egger and inverse-variance weighted (IVW) method. (D) Leave-one-out sensitivity analysis for causal estimates of cosmetic mammoplasty on neuroticism. The red lines represent the overall analytical results of IVW. SNP: single nucleotide polymorphism.


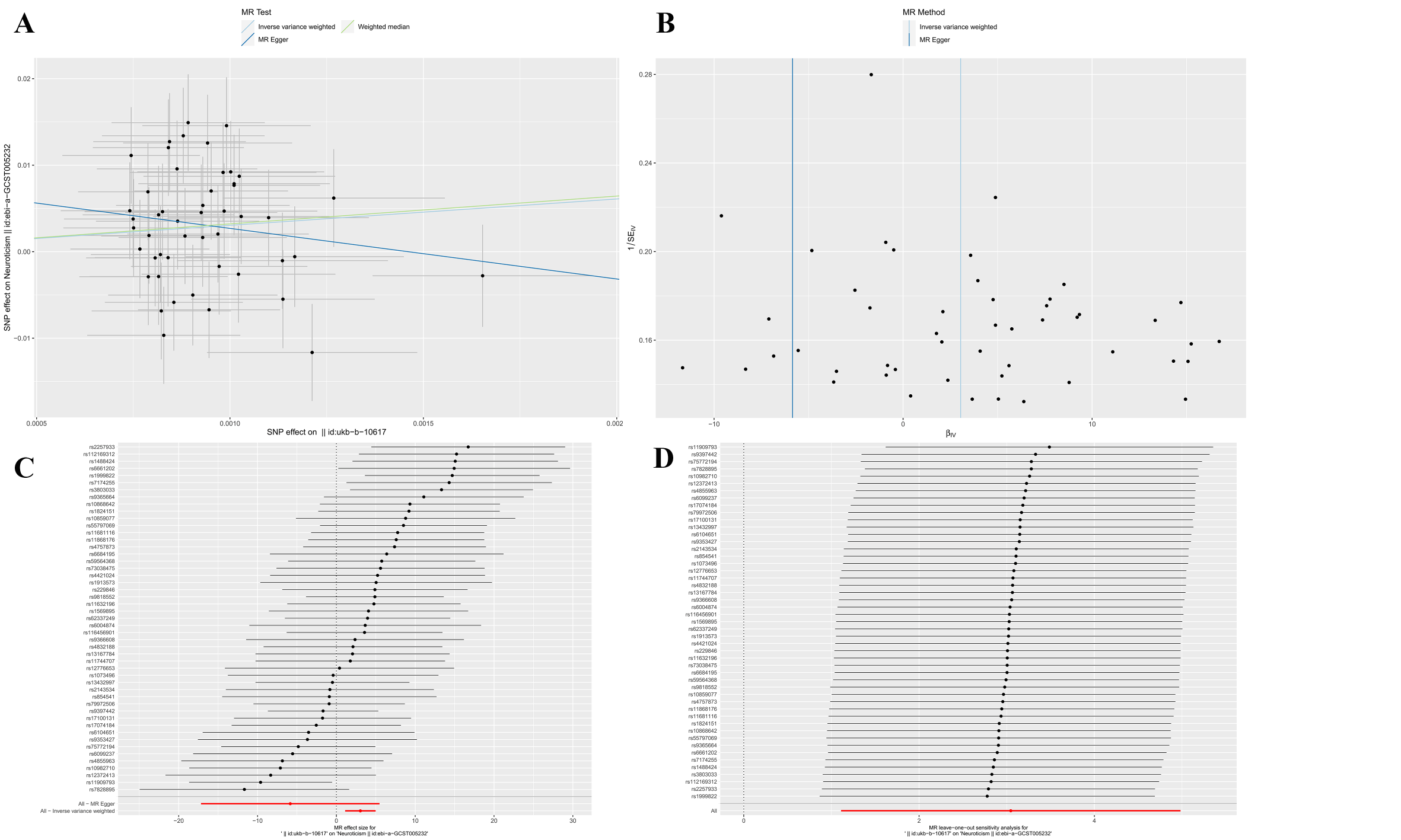

Supplement: Supplementary file 2 [file medi-105-e47580-s002.docx]
